# District-level monitoring of universal health coverage, India

**DOI:** 10.2471/BLT.23.290854

**Published:** 2024-06-25

**Authors:** Arnab Mukherji, Megha Rao, Sapna Desai, S V Subramanian, Gagandeep Kang, Vikram Patel

**Affiliations:** a Centre for Public Policy, Indian Institute of Management, IIM Bangalore, Bengaluru, Karnataka 560076, India.; b Centre for Health Economics, University of York, York, England.; c Population Council Institute, New Delhi, India.; d Harvard Center for Population and Development Studies, Cambridge, United States of America (USA).; e Division of Gastrointestinal Sciences, Christian Medical College, Vellore, India.; f Department of Global Health and Social Medicine, Harvard Medical School, Cambridge, USA.

## Abstract

**Objective:**

To develop a framework and index for measuring universal health coverage (UHC) at the district level in India and to assess progress towards UHC in the districts.

**Methods:**

We adapted the framework of the World Health Organization and World Bank to develop a district-level UHC index (UHC*_d_*). We used routinely collected health survey and programme data in India to calculate UHC*_d_* for 687 districts from geometric means of 24 tracer indicators in five tracer domains: reproductive, maternal, newborn and child health; infectious diseases; noncommunicable diseases; service capacity and access; and financial risk protection. UHC*_d_* is on a scale of 0% to 100%, with higher scores indicating better performance. We also assessed the degree of inequality within districts using a subset of 14 tracer indicators. The disadvantaged subgroups were based on four inequality dimensions: wealth quintile, urban–rural location, religion and social group.

**Findings:**

The median UHC*_d_* was 43.9% (range: 26.4 to 69.4). Substantial geographical differences existed, with districts in southern states having higher UHC*_d_* than elsewhere in India. Service coverage indicator levels were greater than 60%, except for noncommunicable diseases and for service capacity and access. Health insurance coverage was limited, with about 10% of the population facing catastrophic and impoverishing health expenditure. Substantial wealth-based disparities in UHC were seen within districts.

**Conclusion:**

Our study shows that UHC can be measured at the local level and can help national and subnational government develop prioritization frameworks by identifying health-care delivery and geographic hotspots where limited progress towards UHC is being made.

## Résumé

**Objectif:**

Élaborer un cadre et un indice servant à mesurer la couverture sanitaire universelle (CSU) à l'échelle des districts en Inde, et évaluer les progrès effectués dans ce domaine.

**Méthodes:**

Nous avons adapté le cadre de l'Organisation mondiale de la Santé et de la Banque mondiale pour mettre au point un indice de CSU par district (CSU*_d_*). Nous avons utilisé des données recueillies systématiquement dans le cadre d'un programme et d'une enquête sur la santé en Inde pour calculer le CSU*_d_* de 687 districts, en partant des moyennes géométriques de 24 indicateurs traçants relevant de cinq catégories: santé reproductive, maternelle, néonatale et infantile; maladies infectieuses; maladies non transmissibles; capacité et accès aux services; et enfin, protection contre les risques financiers. Le CSU*_d_* est compris entre 0 et 100%, les scores les plus élevés indiquant de meilleures performances. Nous avons également évalué le degré d'inégalité au sein des districts à l'aide d'un sous-ensemble de 14 indicateurs traçants. Les sous-groupes défavorisés reposaient sur quatre formes d'inégalité: quintiles de richesse, implantation urbaine ou rurale, groupe religieux et groupe social.

**Résultats:**

Le CSU*_d_* médian était de 43,9% (écart: 26,4 à 69,4). Nous avons observé des variations géographiques considérables, les districts des États du sud affichant un meilleur CSU*_d_* que partout ailleurs en Inde. L'indicateur relatif à la couverture des services dépassait les 60%, sauf pour les maladies non transmissibles ou encore la capacité et l'accès aux services. De son côté, la couverture d'assurance-maladie était limitée, environ 10% de la population étant confrontée à des dépenses de santé catastrophiques entraînant un appauvrissement. Enfin, d'importantes disparités dans la CSU ont été constatées à l'intérieur des districts.

**Conclusion:**

Notre étude montre que la CSU peut être mesurée à l'échelle locale, ce qui peut aider les autorités locales et nationales à hiérarchiser les priorités en identifiant les prestations de soins de santé et les zones géographiques à risque où l'évolution vers une CSU se fait attendre.

## Resumen

**Objetivo:**

Desarrollar un marco y un índice para medir la cobertura sanitaria universal (CSU) a nivel de distrito en India y evaluar los avances hacia la CSU en los distritos.

**Métodos:**

Se adaptó el marco de la Organización Mundial de la Salud y del Banco Mundial para desarrollar un índice de cobertura sanitaria universal a nivel de distrito (CSU*_d_*).Se utilizaron datos de encuestas y programas de salud recopilados sistemáticamente en India para calcular el CSU*_d_* de 687 distritos a partir de medias geométricas de 24 indicadores de seguimiento en cinco ámbitos de seguimiento: salud reproductiva, materna, neonatal e infantil; enfermedades infecciosas; enfermedades no transmisibles; capacidad y acceso a los servicios; y protección contra riesgos financieros. El CSU*_d_* está en una escala de 0 a 100%, y los puntajes más altos indican un mejor desempeño. También se evaluó el grado de desigualdad dentro de los distritos utilizando un subconjunto de 14 indicadores de seguimiento. Los subgrupos desfavorecidos se basaron en cuatro dimensiones de desigualdad: quintil de riqueza, ubicación urbano-rural, religión y grupo social.

**Resultados:**

El promedio de CSU*_d_* fue del 43,9% (rango: 26,4 a 69,4). Existían diferencias geográficas significativas: los distritos de los estados del sur tenían un CSU*_d_* más alto que los del resto de India. Los niveles de los indicadores de cobertura de servicios eran superiores al 60%, excepto para las enfermedades no transmisibles y para la capacidad y el acceso a los servicios. La cobertura del seguro sanitario era limitada, y alrededor del 10% de la población se enfrentaba a gastos sanitarios catastróficos y empobrecedores. Se observaron disparidades significativas en la CSU según el nivel de riqueza dentro de los distritos.

**Conclusión:**

Este estudio demuestra que la CSU puede medirse a escala local y puede ayudar a los gobiernos nacionales y subnacionales a desarrollar marcos de priorización mediante la identificación de los puntos críticos geográficos y de prestación de asistencia sanitaria en los que los avances hacia la CSU son limitados.

## ملخص

**الغرض:**

تطوير إطار عمل مؤشر لقياس التغطية الصحية الشاملة (UHC) على مستوى المناطق في الهند، وتقييم التقدم المحرز نحو التغطية الصحية الشاملة في المناطق.

**الطريقة:**

قمنا بضبط إطار عمل كل من منظمة الصحة العالمية والبنك الدولي، لتطوير مؤشر التغطية الصحية الشاملة (UHC *_d _* على مستوى المناطق). قمنا باستخدام بيانات وبرنامج المسح الصحي، والتي تم جمعها بشكل روتيني في الهند لحساب مؤشر التغطية الصحية الشاملة لعدد 687 منطقة من المتوسط الهندسي لعدد 24 مؤشر تتبع في خمسة نطاقات للتتبع: الصحة الإنجابية، وصحة الأم، وصحة حديثي الولادة، وصحة الطفل؛ والأمراض المعدية؛ الأمراض غير المعدية؛ القدرة على تقديم الخدمة والحصول إليها؛ والحماية من المخاطر المالية. يتم تقييم مؤشر التغطية الصحية الشاملة على مقياس من 0 إلى %100، وتشير الدرجات الأعلى إلى أداء أفضل. كما قمنا أيضًا بتقييم درجة عدم المساواة داخل المناطق باستخدام مجموعة فرعية مكونة من 14 مؤشرًا للتتبع. واستندت المجموعات الفرعية المهمشة إلى أربعة أبعاد لعدم المساواة: فئة الثروة، والموقع الحضري والريفي، والدين، والطبقة الاجتماعية.

**النتائج:**

كان مؤشر التغطية الصحية الشاملة (النطاق: 26.4 إلى 69.4). تواجدت اختلافات جغرافية أساسية، حيث تتمتع المناطق في الولايات الجنوبية بمؤشر للتغطية الصحية الشاملة أعلى من الأماكن الأخرى في الهند. وكانت مستويات مؤشر تغطية الخدمة أكبر من %60، فيما عدا الأمراض غير المعدية، والقدرة على تقديم الخدمة وإمكانية الحصول إليها. كانت تغطية التأمين الصحي محدودة، إذ يواجه نحو %10 من السكان نفقات صحية باهظة ومكلفة. ولوحظت فوارق كبيرة على أساس الثروة في التغطية الصحية الشاملة داخل المناطق.

**الاستنتاج:**

تظهر دراستنا أنه يمكن قياس التغطية الصحية الشاملة على المستوى المحلي، ويمكن أن تساعد الحكومة الوطنية والإقليمية على تطوير أطر عمل للأولويات من خلال تحديد النقاط الساخنة الجغرافية لتقديم الرعاية الصحية، حيث يتم إحراز تقدم محدود نحو التغطية الصحية الشاملة.

## 摘要

**目的:**

旨在制定一个用于衡量印度县级全民健康覆盖 (UHC) 情况的框架和指数，并评估各个县在实现 UHC 方面的进展情况。

**方法:**

通过借鉴世界卫生组织和世界银行的框架，我们制定了一个县级 UHC 指数 (UHCd)。我们使用在印度收集的常规健康调查和规划数据，根据以下五大示踪领域的 24 个示踪指标的几何平均值计算出了 687 个县的 UHCd：生殖、孕产妇、新生儿和儿童健康；传染病；非传染性疾病；服务能力与其可及性；以及金融风险保护。UHCd 的评分范围为 0 到 100%，分数越高表明表现越好。我们还使用其中 14 个示踪指标评估了各个县的不平等程度。对弱势亚群体的评估基于四个不平等维度：财富五分位数、城乡位置、宗教信仰和社会群体。

**结果:**

UHCd 中位数为 43.9%（范围：26.4 - 69.4）。存在巨大的地理差异，印度南部各邦下属县的 UHCd 明显高于其他县。除非传染性疾病和服务能力与其可及性领域外，服务覆盖率指标水平均高于 60%。健康保险覆盖范围有限，约 10% 的人口面临着灾难性和致贫性卫生支出。在推行 UHC 的过程中，我们发现各县存在巨大的财富差异。

**结论:**

研究表明，我们可以在地方层面对 UHC 情况进行衡量，并且可以通过锁定在实现 UHC 方面进展有限的卫生保健服务和地理集中区域，帮助国家和地方政府制定确定优先次序的框架。

## Резюме

**Цель:**

Разработать систему и индекс для измерения всеобщего охвата населения услугами здравоохранения (ВОУЗ) на уровне округа в Индии и оценить прогресс в достижении ВОУЗ в округах.

**Методы:**

Для разработки индекса ВОУЗ на уровне округов (UHC*_d_*) использовалась система Всемирной организации здравоохранения и Всемирного банка. Для расчета показателя UHC*_d_* для 687 округов Индии использовались данные регулярных обследований и программ в области здравоохранения, полученные на основе геометрических средних значений 24 показателей в пяти областях: репродуктивное здоровье, здоровье матери, новорожденного и ребенка; инфекционные заболевания; неинфекционные заболевания; возможность предоставления услуг и доступ к ним; защита от финансовых рисков. Показатель UHC*_d_* оценивается по шкале от 0 до 100%, при этом более высокие баллы свидетельствуют о лучшей результативности. Кроме того, проведена оценка степени неравенства внутри округов с использованием подмножества из 14 отслеживаемых показателей. Неблагополучные подгруппы были определены по четырем параметрам неравенства: квинтиль благосостояния, городская и сельская местность, религия и социальная группа.

**Результаты:**

Медиана UHC*_d_* составила 43,9% (диапазон: от 26,4 до 69,4). Существовали значительные географические различия: в округах южных штатов показатель UHC*_d_* был выше по сравнению с другими регионами Индии. Уровни показателей охвата услугами превышали 60%, за исключением неинфекционных заболеваний, а также возможностей предоставления услуг и доступа к ним. Охват услугами медицинского страхования был ограничен, и около 10% населения сталкивались с катастрофическими и приводящими к обнищанию расходами на здравоохранение. Внутри округов наблюдалось значительное неравенство по уровню благосостояния в отношении всеобщего охвата услугами здравоохранения.

**Вывод:**

Результаты исследования свидетельствуют о том, что всеобщий охват населения услугами здравоохранения может быть измерен на местном уровне и может помочь национальным и субнациональным органам власти разработать систему приоритетов, определив систему оказания медицинских услуг и географические точки, в которых прогресс на пути к всеобщему охвату населения услугами здравоохранения является ограниченным.

## Introduction

Universal health coverage (UHC) has emerged as a major goal in global health within the post-2015 millennium development agenda.^1^ The objectives of UHC are typically defined by three dimensions: the population that is covered by pooled funds; the proportion of direct health costs covered by pooled funds; and the health services covered by those funds.^2^ While these dimensions provide a framework for a country’s aspirations for its health system, the path to UHC varies from one country to another. Over the past four decades, many low- and middle-income countries, including India, have implemented health-sector reforms in pursuit of UHC. Most of these reforms have embraced some degree of health system decentralization, primarily due to the adoption of decentralized governance in public services.^3^ Decentralization is a sociopolitical process that transfers authority and responsibility in planning, management and decision-making from central government to local authorities.^4^^–^^6^ In most decentralized governance systems, the responsibility of health-care delivery is shared between the national and provincial (or state) governments, forming a two-tier system.^7^ This structure has led to the development of national and subnational UHC indexes to monitor progress.^8^^–^^17^ What is relatively uncommon is a three-tier arrangement, where exclusive power is granted to local governments below the provincial (state) government.^18^ The complexity of three-tier decentralization provides a compelling rationale for parallel geographic disaggregation of the UHC index. This approach aims to track progress in greater detail, acknowledging the distinct challenges and dynamics at each level of governance. Moreover, the resurgence of interest in Alma Ata principles, which emphasize the importance of local health systems as an essential element to achieve UHC,^19^ calls for extending metrics to the local level to gain insight on how a country can move its health system towards UHC more equitably.

Many low- and middle-income countries, for example Argentina, Brazil,^20^ India,^6^ Indonesia,^7^ Nigeria,^20^ Pakistan,^21^ Philippines,^22^ South Africa^23^ and Uganda,^22^ have adopted a three-tier decentralized system. Each of these countries has, to varying degrees, decentralized the design and implementation of UHC programmes to the third tier, namely local governments. Of these countries, India has a unique position as it accounts for a sixth of the world’s population, a fifth of the global burden of disease and a quarter of all households facing catastrophic health expenditure. As a result, it is perhaps the country for which attaining sustainable development goal (SDG) 3.8, i.e. achieving UHC, is particularly important.^24^ Additionally, districts, which constitute local governments in India, have an average population of about 1.9 million.^24^ These factors make India well-suited to take UHC metrics to the local government level to support more precise decision-making.

While the Indian government has created composite measures to track health-system performance at the state level, we lack comprehensive district-level measures, which is a challenge for decentralization efforts. Identifying which districts are closest or furthest from achieving UHC is difficult without district-level UHC measures. In addition, given the multidimensional nature of UHC which spans health-care services and financial risk protection, such an index can identify where a district must focus its efforts. Thus, a district-level UHC index, which we call UHC*_d_*, has the potential to identify geographic hotspots of low UHC, help design appropriate district-level programmes, and enable progress in UHC in districts to be explicitly linked to public expenditure and outcomes.

The aim of this study was to outline a framework for measuring UHC at the district level in India and provide an adaptable method to monitor progress at local levels in three-tier decentralized systems.

## Methods

### Study design

Among the various UHC measurement frameworks available, we chose to base our UHC index on the framework developed by the World Health Organization (WHO) and the World Bank.^25^ This framework, which is used to produce the global monitoring reports that track UHC progress in 183 countries, follows methods proposed in 2018.^8^ We adapted this framework to the district level and used routinely collected health surveys and programme data in India to compare the district health systems.

Following the WHO and World Bank terminology, UHC*_d_* has five tracer domains: reproductive, maternal, newborn and child health; infectious diseases; noncommunicable diseases; service capacity and access; and financial risk protection. In line with earlier research, we combined service coverage and financial protection into one measure to capture both the unmet need for services and the resultant economic burden of unmet needs.^10^^,^^26^ Table 1 (available at https://www.who.int/publications/journals/bulletin). lists each tracer domain, which consists of a set of tracer areas; each tracer area is composed of tracer indicators and collectively, we have 24 district-level tracer indicators capturing information on service delivery and contextual factors relevant to tracking performance.

**Table 1:** Sources of data and list of tracer indicators, areas and domains

Tracer domain, area and indicator	Comparison with WHO and World Bank indicators^8^^,^^25^
**Reproductive, maternal, new-born, and child health^a^**	
*Pregnancy and delivery care*	
% of mothers reporting at least four antenatal care visit	Identical
% of mothers who received postnatal care from skilled health personnel within 2 days of delivery	Additional
% of live births attended by skilled health personnel	Additional
% of births occurring in health facilities, or institutional deliveries	Additional
*Family planning*	
% of women aged 15–49 years who are married or in a union using modern contraception methods	Identical
*Full immunization*	
% of children aged 12–23 months who have received BCG and measles vaccines, and three doses each of polio and DPT vaccines	Modified
**Infectious diseases**	
*Water, sanitation and hygiene^a^*	
% of the population using an improved sanitation facility	Identical
% of the population using an improved drinking-water source	Additional
*Effective treatment of tuberculosis^b^*	
The treatment success rate (%) for new pulmonary smear-positive tuberculosis cases	Modified
**Noncommunicable diseases^a^**	
*Prevention of cardiovascular diseases*	
% of adults aged 18 years and older with systolic blood pressure < 140 mmHg and diastolic blood pressure < 90 mmHg (regardless of treatment status)	Identical
*Prevention of diabetes*	
% of adults aged 18 and older with blood glucose (sugar) ≤ 140 mg/dL (regardless of treatment status)	Modified
*Tobacco control and tobacco use*	
% of adults aged 15 years and older not smoking tobacco	Modified
*Cancer detection and treatment*	
% of women who have ever undergone a screening test for cervical cancer	Identical
% of women who have ever undergone a screening test for breast cancer	Additional
**Service capacity and access^c,d^**	
*Health facility access*	
% of functioning facilities available in a district providing round-the-clock health care as per norms of Indian Public Health Standards	Additional
*Health infrastructure*	
Beds available in a district as a % of the norm specified by the Indian Public Health Standards	Modified
*Essential medicines*	
% of essential medicines (out of the list of the Indian Public Health Standards) available in primary health-care centres and community health centres in a district	Modified
*Health workforce*	
Doctors available in a district as a % of the norm specified by the Indian Public Health Standards	Modified
Paramedical staff available in a district as a % of the norm specified by the Indian Public Health Standards	Additional
*Health personnel with maternal and child health training*	
% of health personnel in a district who received training in skilled attendance at birth in the past 5 years	Additional
% of health personnel in a district who received training in basic emergency obstetric care in the past 5 years	Additional
**Financial risk protection**	
*Health insurance coverage^a^*	
% of the population in a district covered by a health insurance scheme	Additional
*Protection from catastrophic health expenditure^e^*	
% of the population in a district not experiencing catastrophic health expenditure	Modified
*Protection from impoverishment^e^*	
% of the population in a district not experiencing impoverishment due to health expenses	Additional

BCG: bacille Calmette–Guerin; DPT: diphtheria, pertussis, tetanus; WHO: World Health Organization.

^a^ Data source: National Family Health Survey (2019–2021).^27^

^b^ Data source: National tuberculosis report (2014).^28^

^c^ Data source: District Level Health Survey (2012–2013).^29^

^d^ For detailed estimation process, see technical report in online repository.^30^

^e^ Data source: National Sample Survey Office survey 75th round (2017–2018).^31^

### Variables

Our UHC index contains a wider set of indicators than the core indicators in the WHO and World Bank framework. Thirteen of the 24 tracer indicators are based on the WHO and World Bank framework, while we included 11 additional variables to capture district-level UHC priorities in India (Table 1). From the original set of WHO and World Bank indicators, we measured five in an identical way. We measured the rest of the indicators differently given data constraints and relevance (Table 1). We split health workforce into two tracer indicators (Table 1), unlike the WHO and World Bank framework. The detailed list of tracer indicators, related changes, estimation method and the rationale are summarized in the technical report available in the online repository.^30^ Where data were unavailable, we used proxy indicators, especially for noncommunicable diseases and service capacity and access (online repository).^30^ To allow stable estimation at the district level, we excluded four tracer indicators used in the original WHO and World Bank framework: malaria prevention; treatment of human immunodeficiency virus; care seeking for symptoms of pneumonia; and health security, due to the lack of data in the surveys we reviewed.

### Data sources and measurement

We primarily used four publicly available secondary data sources. Most indicators in the domains of reproductive, maternal, newborn and child health, infectious diseases, and noncommunicable diseases (except tuberculosis treatment) were drawn from the fifth round of the National Family and Health Survey (2019–2021).^27^ This survey provides district-level estimates for family planning, reproductive and child health, and noncommunicable diseases for all 707 districts and 36 states and union territories of India. The district-level estimates of tuberculosis treatment success rates were from the 2014 annual tuberculosis report.^28^ We took information on service capacity and access from the population-linked facility module of the District-Level Household Survey (2012–2013).^29^ This survey contains information on human resources, infrastructure and services for a sample of subcentres and primary health-care centres designed to be representative at the district level and a census of community health centres, and district and subdistrict hospitals. Although the information is from 2012–2013, this survey provides the most recent district-level facility data. We focused only on primary health-care centres and community health centres since they serve as the people’s first point of contact with qualified doctors in the districts. In addition, instead of using the WHO benchmarks and list of core medicines to estimate indicators related to service capacity and access, we used the minimum thresholds set by the Indian Public Health Standards (online repository).^30^ Data for catastrophic health expenditure and impoverishment came from the 75th round of the health survey of the National Sample Survey Office,^31^ which has nationally representative health expenditure data. Our choice of data sources reflects: (i) district representability; (ii) high response rates; and (iii) availability of multiple measures relevant to UHC*_d_*. We transformed all tracer indicators such that 0 indicates no coverage while 100 indicates complete coverage for the population. For financial risk protection, we took the complement of the incidence of impoverishment and catastrophic health expenditure, i.e. the percentage of the population not incurring catastrophic and impoverishing payments to match the directionality of UHC*_d_*.

To maximize geographical coverage, we applied imputation techniques to generate estimates for districts with missing data. When faced with missing data due to geographic redistricting over time, we followed the imputation method in a 2021 publication.^32^ We used the STATA (StataCorp. LP, College Station, United States of America) hot-deck imputation algorithm^33^ for districts lacking data on indicators of service capacity and access. Additionally, we used regression imputation^34^ to estimate values for catastrophic health expenditure and impoverishment for all districts in the data set (online repository).^30^ Consequently, the UHC*_d_* was constructed for 687 of 707 districts across India.

### Index construction

**Fig. 1:**
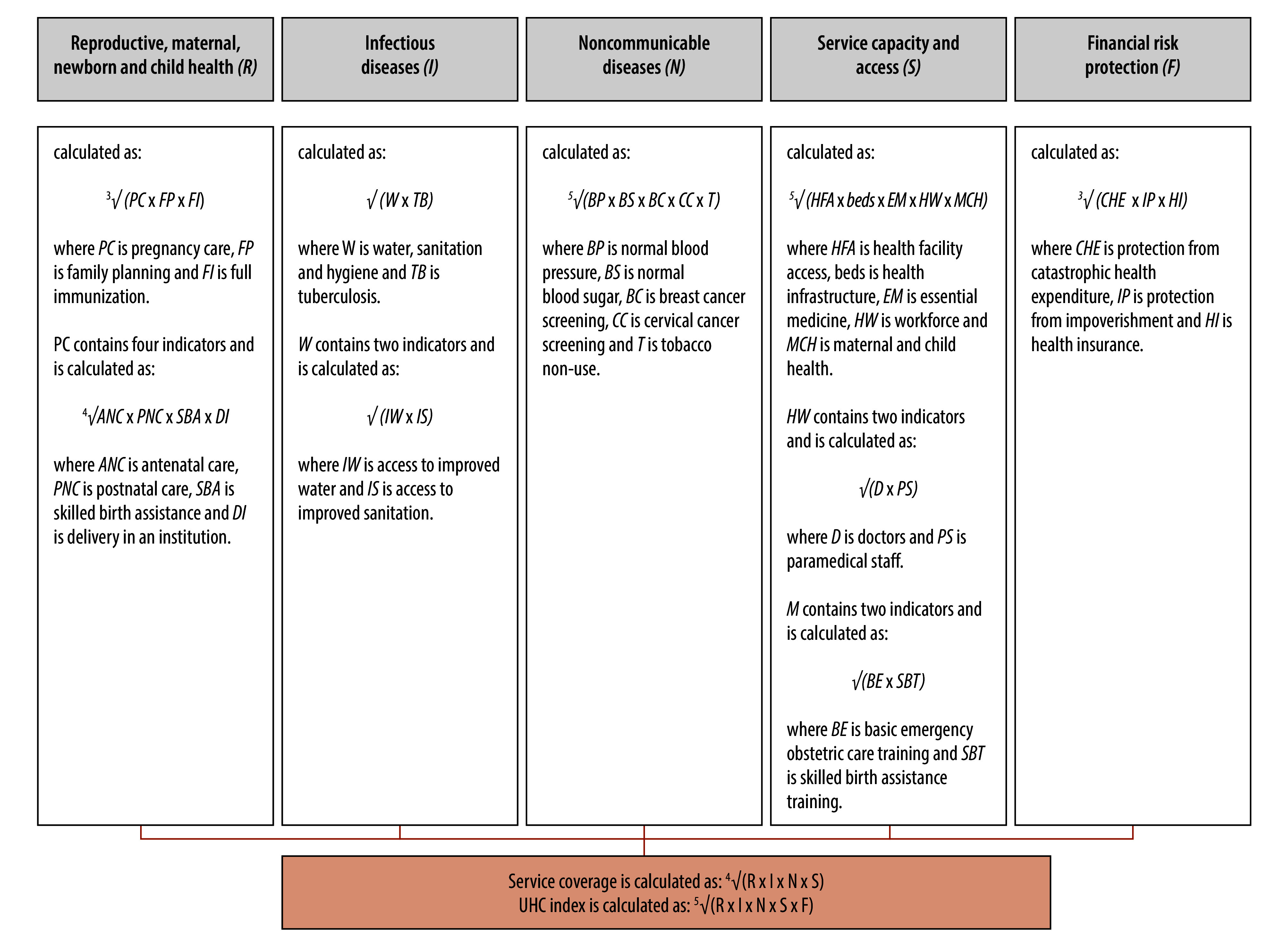
Method used to derive district-level universal health coverage values, India

We used geometric means to aggregate tracer indicators, ensuring equal weighting for each tracer indicator. This method, chosen for its resilience to extreme values, is consistent with established practices.^8^^,^^10^^,^^14^^,^^17^ To calculate UHC*_d_*, we initially aggregated within a tracer area by calculating the geometric mean of the constituent tracer indicators. Then, we aggregated within a tracer domain by calculating the geometric mean of the constituent tracer areas. Finally, we derived the UHC*_d_* for a district by calculating the geometric mean across the five tracer domains (Fig. 1; available at https://www.who.int/publications/journals/bulletin). Additionally, UHC*_d_* can be disaggregated into its two key components: (i) service coverage, calculated as the geometric mean of reproductive, maternal, newborn and child health, infectious diseases, noncommunicable diseases, service capacity and access; and (ii) financial risk protection. Since we measured all tracer indicators on a scale of 0% to 100%, the UHC*_d_* is on the same scale, with higher scores indicating better performance.

UHC: universal health coverage.

Note: variables are explained in Table 1.

### Statistical analysis

Districts with comparable (high or low) UHC*_d_* were clustered together in certain states. We used a multilevel model with only intercept and state effects to quantify UHC*_d_* variance between and within states. Additionally, in low- and middle-income countries, studies have shown that higher levels of UHC are associated with lower levels of poverty.^15^^,^^17^^,^^35^ In our study, we further investigated the relationship between UHC and poverty by using the multidimensional poverty index (online repository).^30^^,^^36^ Unlike conventional income-based measures, the multidimensional poverty index captures a slightly overlapping but largely distinct aspect of poverty, that is, it identifies non-income factors associated with social inequalities.^37^ Moreover, this index is also a recognized metric in India for reporting on poverty.

### Equity

We also summarized the degree of inequality within districts using a subset of 14 tracer indicators (online repository).^30^ Following a 2018 study,^8^ we compared the UHC*_d_* of the district population to that of the historically disadvantaged population groups. This comparison ensured consistency with the SDG focus on national coverage and coverage in the most disadvantaged groups.^8^ The disadvantaged population subgroups were based on four key inequality dimensions: wealth quintile, urban–rural location, religion and social group. Specifically, for each district, we calculated the ratio of the geometric mean of the tracer indicators for the disadvantaged subgroup to the geometric mean for the district. We then multiplied this ratio with UHC*_d_*, as calculated for the district, to give the value of UHC*_d_* for the subgroup.

### Sensitivity analysis

To test the robustness of our results, we recalculated and ranked the districts using different alternative approaches. These approaches included: adjusting the UHC*_d_* for inequality in intervention coverage between the poor and better-off districts by switching from the population mean to the so-called achievement index;^38^ recalculating UHC*_d_* using arithmetic means and overall geometric mean instead; and assessing the index’s sensitivity to the choice of tracer indicators by dropping indicators one at a time. We determined sensitivity by estimating the Spearman rank correlation between the original district rankings and the rankings based on alternative approaches. Finally, using state-level rural health statistics of 2020–2021 instead of the district-specific data from the District-Level Household Survey (2012–2013), we confirmed the robustness of our findings.

## Results

Overall, 97.2% (687/707) of the districts had information on all 24 tracer indicators. The median value of UHC*_d_* was 44.0% (Table 2), ranging from 26.4% in Baksa (Assam) to 69.4% in the Nilgiris (Tamil Nadu). Only 21.1% (145/687) of the districts had a UHC*_d_* value greater than 50.0% (online repository).^30^ The districts had a median service coverage of 40.2% and 66.6% for financial risk protection (Table 2). We categorized the districts into high, medium and low UHC*_d_* values and calculated the median values for UHC*_d_* and other tracer domains by terciles (online repository).^30^

**Table 2:** Summary statistics for district-level universal health coverage index by tracer domains, India

Measures	UHC*_d_*	Service coverage	Reproductive, maternal, newborn, and child health	Infectious diseases	Noncommunicable diseases	Service capacity and access	Financial risk and protection
No. of districts	687	687	707	705	707	687	707
Median, %	44.0	40.2	64.8	82.8	9.8	54.9	66.6
IQR, %	40.0–48.3	35.7–44.4	54.5–73.2	78.7–86.7	7.8–13.1	41.0–66.8	54.1–79.0
SD, %	7.1	7.1	12.7	7.7	5.2	16.2	15.1
Min, %	26.4	19.8	18.8	8.8	4.3	9.5	23.9
Max, %	69.4	67.3	86.0	97.8	37.1	92.6	96.3

IQR: interquartile range; max: maximum; min: minimum; SD: standard deviation; UHC*_d_*: district-level universal health coverage index.

We also disaggregated UHC*_d_* by tracer areas and tracer indicators (online repository).^30^ Infectious diseases had the highest median coverage (82.8%; range: 8.8 to 97.8) across all tracer domains. Within infectious diseases, coverage for tuberculosis treatment was 88.0%; and water, sanitation and hygiene was 79.4%. Noncommunicable diseases had the lowest coverage (9.8%; range: 4.3 to 37.1), with more than half of the districts having a coverage of only 5.0% to 10.0%. Cancer screening coverage was poor (< 1.0%). However, coverage for prevention of hypertension (63.2%) and diabetes treatment (92.6%) and reduction in tobacco use (92.6%) was high at the district level. 

The median reproductive, maternal, newborn and child health coverage was 64.8%, ranging from 18.8% to 86.0%. Within this domain, family planning had the least coverage (55.6%), followed by immunization (63.4%), while coverage of delivery care was 80.1%. The overall service capacity and access availability was 54.9%, ranging from 9.5% to 92.6%, with most of its tracer areas performing poorly. Only 48.7% of the district populations had access to round-the-clock health facilities. UHC*_d_* for availability of health personnel was 49.4%, and for availability of essential medicines it was 60.0%. Maternal and child health training for health personnel at the district level was only 48.0%. The only indicator performing well in this domain was availability of beds (87.9%). Financial risk protection coverage at the district level ranged from 23.9% to 96.3% with a median of 66.6%. Health insurance coverage was 40.7%, and 8.0% of the district population experienced catastrophic health expenditure, with 11.2% facing impoverishment due to health-care spending.

Significant spatial differences in UHC*_d_* existed across states and regions in India (online repository).^30^ Districts in southern states such as Andhra Pradesh, Kerala, Puducherry and Tamil Nadu had higher coverage (> 50%) compared with the relatively less-developed states from the central, eastern and north-eastern regions such as Bihar, Jharkhand, Manipur and Uttar Pradesh (< 40%). Distribution based on level of UHC*_d_* (low, medium or high) showed similar findings (online repository).^30^ Most high-performing districts were from the southern states of Andhra Pradesh, Kerala and Tamil Nadu, and all districts in Andhra Pradesh and Tamil Nadu were high performers; in fact, the top three performing districts were from Tamil Nadu (Table 3). On the other hand, many districts from central and eastern states such as Bihar, Jharkhand and Uttar Pradesh were poor performers. All districts in Manipur and 89.3% (67/75) of the districts in Uttar Pradesh were low performers. Eastern states, such as Bihar, Jharkhand and Odisha, also had a considerable number of low performers. A few states, such as Chhattisgarh, Madhya Pradesh and Meghalaya, had a mix of well and poorly performing districts.

**Table 3:** District-level universal health coverage index, by state, India

State, by region	Total no. of districts	Median UHC*_d_*, % (IQR)	No. of districts categorized by UHC*_d_* index as:		
			High	Medium	Low
**Central**					
Chhattisgarh	27	46.0 (41.6–47.4)	9	14	4
Madhya Pradesh	51	46.4 (42.9–48.5)	25	18	8
Uttar Pradesh	75	37.3 (34.7–40.3)	0	8	67
Uttarakhand	13	47.4 (43.7–51.1	9	3	1
**Eastern**					
Andaman and Nicobar	3	46.4 (42.2–50.6)	1	2	0
Bihar	38	38.3 (35.9–41.2)	0	8	30
Jharkhand	24	38.8 (35.6–40.2)	0	3	21
Odisha	30	40.5 (38.0–42.3)	0	9	21
Sikkim	4	42.4 (41.9–44.3)	0	4	0
West Bengal	19	41.4 (39.7–44.7)	3	8	8
**North-eastern**					
Arunachal Pradesh	20	41.6 (38.1–42.9)	0	10	10
Assam	33	40.9 (39.4–42.9)	1	15	17
Manipur	9	37.5 (35.4–39.0)	0	0	9
Meghalaya	11	42.6 (41.4–46.1)	1	8	2
Mizoram	8	54.4 (48.1–56.4)	7	1	0
Nagaland	11	39.0 (37.3–43.4)	0	3	8
Tripura	8	43.1 (41.9–45.1)	0	7	1
Chandigarh	1	49.2 (49.2–49.2)	1	0	0
Haryana	22	44.9 (43.9–48.4)	8	12	2
Himachal Pradesh	12	45.2 (42.9–46.8)	4	8	0
Jammu and Kashmir	20	40.9 (39.6–42.8)	1	7	12
Ladakh	2	43.1 (42.5–43.7)	0	2	0
Punjab	22	46.5 (44.2–49.0)	10	11	1
Rajasthan	33	48.2 (45.0–51.4)	19	14	0
**Southern**					
Andhra Pradesh	13	53.5 (50.5–58.3)	13	0	0
Karnataka	30	46.5 (44.9–48.8)	12	17	1
Kerala	14	49.9 (46.7–53.4)	11	2	1
Puducherry	4	55.5 (49.7–59.5)	3	1	0
Tamil Nadu	31	63.0 (59.6–67.4)	31	0	0
Telangana	30	52.9 (51.5–54.1)	29	1	0
**Western**					
Goa	2	52.2 (49.7–54.6)	2	0	0
Gujarat	33	44.7 (42.2–46.8)	9	20	4
Maharashtra	34	47.2 (45.8–48.9)	20	13	1
**Total**	**687**	**43.96 (40.03–48.25)**	**229**	**229**	**229**

IQR: interquartile range; UHC*_d_*: district-level universal health coverage index.

**Fig. 2:**
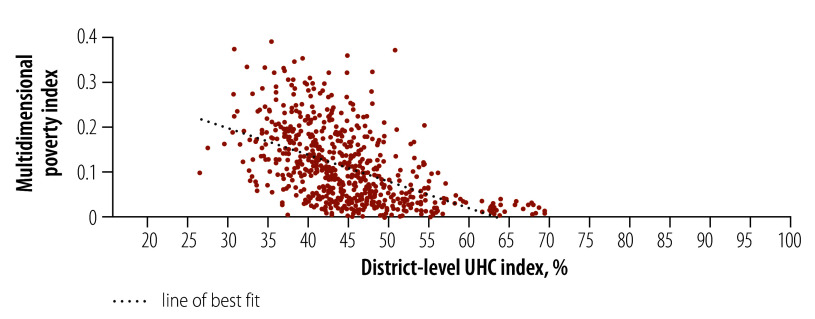
Correlation between district-level universal health coverage index and multidimensional poverty index at the district level, India

Most variation (69.4%) in UHC*_d_* was due to differences between states (Table 4). The remaining 30.6% was attributable to differences between districts (within-state). UHC*_d_* levels were most homogenous in Sikkim (coefficient of variation < 5.0%), while both Kerala and Puducherry had the highest within-state heterogeneity in UHC*_d_* (coefficient of variation > 12.0% to 14.0%; online repository).^30^ The correlational analysis between UHC*_d_* and the multidimensional poverty index showed a significant negative correlation (coefficient of variation −0.51, *P* < 0.05; Fig. 2). 

**Table 4:** Null model with only state-level effects for district-level universal health coverage index, India

Random effects parameters	Variance, % (SE; 95% CI)	Variance attributable, %
Between states	31.5 (8.05; 19.1–52.0)	69.4
Within state	13.9 (0.77; 12.5–15.5)	30.6

CI: confidence interval; SE: standard error.

UHC: universal health coverage.

Our equity assessment showed that progress in UHC*_d_* was unequal across the different subgroups (online repository).^30^ Wealth-related disparity was the most notable compared with other dimensions. Across the 687 districts, the difference in UHC*_d_* between the poorest quintile and the district population varied from −3.0% to 24.0%. The median UHC*_d_* was lower in the poorest wealth quintile (36.9%) than the district population (43.9%). The UHC*_d_* of subgroups also varied considerably between districts across states.

The UHC*_d_* was stable in various sensitivity tests (online repository).^30^ The district rankings were not sensitive to inequality adjustments (correlation coefficient, *ρ*: 0.9916; *P* < 0.0001). The biggest effect in terms of district rankings was switching to the arithmetic mean as the choice of summary method (*ρ*: 0.8584; *P* < 0.0001). Comparatively, the changes in the rankings were minimal because of using an overall geometric mean method (*ρ*: 0·9614; *P* < 0.0001). As regards sensitivity of the index to the choice of tracer indicator, the Spearman rank correlation was high across all permutations (lowest *ρ*: 0.95). The most pronounced effect on the rankings was when we dropped indicators on cancer screening and health insurance, compared with other indicators. Lastly, we saw a high correlation between recent state-level data and older district-level data on service capacity and access (online repository).^30^

## Discussion

Globally, attention to the governance dimension of UHC and the SDGs, including the role of local governments, is increasing.^39^^–^^41^ Using the WHO and World Bank framework, we developed a district-level UHC index tailored to India’s UHC priorities and context, primarily using nationally representative surveys. This index provides a detailed measure of local UHC that is closer to policy and programme implementation and yet is within a common empirical structure to support comparison across place and time. The index reflects recent research that emphasizes the importance of examining subnational variation in health progress.^14^^,^^42^^,^^43^ Our index also extends the relevance of UHC metrics to encompass three-tier governance systems seen in several low- and middle-income countries. Thus, our proposed UHC framework represents a decentralized and people-centred approach to UHC. Anchored in the WHO and World Bank framework, the index offers a well-defined yet flexible approach for measuring UHC, combining globally consistent and nationally focused data sources.

Our proof-of-concept analysis suggests that UHC varies widely in India: southern districts are, on average, closer to attaining UHC than elsewhere in India. Overall, the majority of Indian districts were unable to reach 50.0% on the UHC index. Service coverage indicator levels were higher than 60.0%, except those indicators related to noncommunicable diseases, and service capacity and access. Health insurance coverage was limited, with about one in 10 people experiencing catastrophic and impoverishing health expenditure. We found evidence of substantial wealth-related disparity in UHC within districts across states, indicating a strong link between poverty and progress towards UHC. Accordingly, different governance, disease incidence,^44^ and social, cultural and political background^45^ imply that India’s pathways to UHC will be largely shaped at the state and district level. Recent research, facilitated by the availability of district-level data in India, highlights the importance of local-level analyses of health outcomes and services, such as in cancer screening,^46^ caesarean sections,^47^ hysterectomies^48^ and health insurance.^49^

With countries adopting UHC as an aspiration for their national policy, studies of several countries have shown varying levels of UHC and different pathways towards achieving UHC.^2^ Preliminary evidence suggests that the challenges to achieving nationwide UHC will depend on tackling inequities and variation in service coverage and financial risk protection within the country.^14^^,^^50^ At a practical level, local-level data and tracking are also essential to inform decision-making and build responsive and resilient health systems. Our UHC*_d_*_,_ provides a single metric for the district-level administration in India, fosters transparent tracking, and enables prioritization of local strategies using publicly available data.

While our conceptualization of UHC*_d_* is easy to implement and facilitates time and cross-district comparisons, certain limitations must be addressed for its continued use. First, since the index is based on routinely collected national surveys – which are not designed with a comprehensive UHC metric in mind – inaccuracies or inconsistencies may emerge over time. These issues may arise due to changing definitions as well as changing needs of what UHC should measure; for example, any UHC metric reflects the health system’s choices on screening and treatment. Deciding where on the path to UHC delivery of care versus a public health function of screening for prevention should be incorporated is important and will need more detailed data and debate.^8^ This need suggests the importance of advocating for data specifically focused on UHC within and across settings. Availability of such data will address the immediate constraints of this analysis, including the reliance on older data for certain indicators and the broader challenge of insufficient district-level health survey and administrative data. Another limitation stems from the lack of district-specific health conditions that are present locally, as we used a nationwide data set. Finally, while our approach supports equity-related analysis, the index does not directly incorporate equity and may understate the strong link between inequality and UHC that we report.

Our work presents a proof-of-concept of how UHC can be measured at the local level of policy implementation rather than at country or policy-design levels. The UHC*_d_* is also a powerful means to identify issues for further, context-specific study. For example, we used the index to identify a range of districts that would be suitable for qualitative research with district-level health system actors as part of the *Lancet* Citizens’ Commission on re-imagining India’s health system. Such research offers the advantage of showing where and how progress towards UHC may be achieved. We believe this research can serve as a starting point for identifying key local policy actions to guide an intensified effort towards UHC over the next decade.
